# A history of cardiovascular magnetic resonance imaging in clinical practice and population science

**DOI:** 10.3389/fcvm.2024.1393896

**Published:** 2024-04-19

**Authors:** Mihir M. Sanghvi, João A. C. Lima, David A. Bluemke, Steffen E. Petersen

**Affiliations:** ^1^ William Harvey Research Institute, Queen Mary University of London, London, United Kingdom; ^2^ Barts Heart Centre, Barts Health NHS Trust, London, United Kingdom; ^3^ Division of Cardiology, Department of Medicine, Johns Hopkins University, Baltimore, MD, United States; ^4^ Department of Radiology, Johns Hopkins University, Baltimore, MD, United States; ^5^ Department of Radiology, University of Wisconsin School of Medicine and Public Heath, Madison, WI, United States

**Keywords:** history of medicine, magnetic resonance imaging, cardiovascular magnetic resonance (CMR) imaging, nuclear magnetic resonance, Multi-Ethnic Study of Atherosclerosis (MESA), UK Biobank

## Abstract

Cardiovascular magnetic resonance (CMR) imaging has become an invaluable clinical and research tool. Starting from the discovery of nuclear magnetic resonance, this article provides a brief overview of the key developments that have led to CMR as it is today, and how it became the modality of choice for large-scale population studies.

## Introduction

Even the most ardent cardiac imager may be forgiven for not having read C. J. Gorter's 1936 manuscript in *Physica* in which he described unsuccessfully attempting to find resonance of lithium-7 nuclei in crystalline lithium fluoride (1). However, it is from here that the story of cardiovascular magnetic resonance (CMR) can trace its origins. Whilst the latest guidelines and research articles make up the bricks of our clinical and academic practice, the mortar—the tales of how these edifices were constructed—can often be even more compelling. In this review article, we embark on a whistlestop tour of the history of CMR in clinical practice and its strides into population science. We hope that state-of-the-art CMR and future directions will be better understood and appreciated when contextualised within the tapestry of innovation and discovery that has preceded it.

## Early NMR research

The phenomenon of nuclear magnetic resonance (NMR) sought by Gorter was discovered independently by Felix Bloch and Edwin Purcell in 1946 (2, 3). They determined that when certain nuclei were placed in a magnetic field and subjected to radiofrequency energy, they absorbed energy and re-emitted this energy when the nuclei returned to their original state. For this discovery, they were awarded the 1952 Nobel Prize in physics. From this point, the field of NMR saw a host of investigations in non-biological and biological samples. The work of Richard Ernst in 1966 resulted in a step change in the field when he demonstrated that it was possible to markedly improve the sensitivity of NMR signals generated by applying the Fourier transformation (4). This mathematical operation, named after the French physicist Jean-Baptiste Joseph Fourier, is now the bedrock upon which magnetic resonance output can be interpreted.

In 1971, Raymond Damadian measured T1 and T2 relaxation times of normal and cancerous *ex vivo* rat tissue, demonstrating that tumours had longer relaxation times (5). However, all NMR experiments until the early 1970's were one-dimensional—no spatial information was available to ascertain where within the sample the NMR signal originated from. The problem of spatial localisation was solved independently by Paul Lauterbur and Peter Mansfield in 1974 (6, 7). They discovered that the addition of a varying magnetic field (a “gradient”) altered the resonance frequency of nuclei in proportion to their position in the magnetic field. By understanding how these gradients could be applied in different orientations, spatial information could be encoded in three dimensions and thereby provide the requisite information for generating an image. Ernst, having heard Lauterbur describe this at a conference in North Carolina in 1974 (8), understood the applicability of his work from NMR spectroscopy and wrote a 1975 publication with Anil Kumar which described a practical method to rapidly reconstruct an image from NMR signals (9). Ernst received the Nobel Prize in chemistry in 1991 and Lauterbur and Mansfield the Nobel Prize in medicine in 2003.

## Towards cardiovascular NMR

With the discovery of spatial localisation, a Rubicon had been crossed. The first anatomical structure imaged in detail was a cross-section of a finger in 1977 by Mansfield and Andrew Maudsley (10). Later the same year, Damadian and colleagues had generated a cross-sectional image of the human chest (11) and by 1978, Hugh Clow and Ian Young had reported the first transverse image of the head (12).

Whilst cardiac structures were visible on these early NMR images, Robert Hawkes and colleagues in Nottingham published results of NMR imaging directed specifically at the heart in 1981 (13). In this manuscript they presciently remarked that electrocardiogram (ECG) gating might improve scan quality and the great potential of cardiovascular NMR given its lack of ionising radiation. By this time, the original term NMR imaging had been replaced by the rather less precise magnetic resonance imaging (MRI) and applications of MRI accelerated rapidly. In 1983, Herfkens et al. published cardiovascular MRI findings at 0.35 Tesla (T) in 244 individuals—a remarkably large sample even by comparison to the size of modern day CMR cohorts (14).

Limitations in early MRI scanners meant the process of generation the NMR signal was slow—to the order of one to several seconds. Due to heart motion during long acquisitions, investigators recognised that synchronisation of the NMR signal to the cardiac cycle was necessary. Higgins and colleagues tested three different triggering methods: peripheral pulse signals, Doppler flow signals and ECG signals. The ECG gating method was demonstrably superior to other methods and became widely adopted in the field.

## Structure and function

Mansfield and his team reported the first real time cine CMR image of a live rabbit heart in 1982 using an echo planar imaging technique they developed (15). CMR was recognised to be useful to provide accurate estimates of cardiac function and left ventricular mass (16) as well as estimating regurgitant fractions in patients with mitral or aortic regurgitation (17). A major disadvantage of early CMR was long acquisition times (e.g., two minutes for each phase of the cardiac cycle). This was solved in the early 1990's by Atkinson and colleagues who introduction segmented data acquisition. With this method, cine CMR image acquisition was accelerated in inverse proportion to the acquired number of frames per cardiac cycle. Importantly, ECG segmented acquisitions combined with fast gradient echo acquisition allowed single breath-hold, two-dimensional cine imaging of the heart (18).

Hawkes, who generated perhaps the first set of dedicated cardiac images using NMR, also worked on the earliest versions of the steady-state free precession imaging sequence (SSFP) in the early 1980's. The SSFP technique temporarily faded in relevance due to its relative inferior image quality compared to the gradient echo imaging techniques. However, by the turn of the millennium, better magnet field homogeneity and improved gradient hardware with rapid switching had remarkably improved image quality. By the late 1990s, the SSFP method was providing far clearer blood pool-to-myocardium and epicardial fat-to-myocardium delineation and is currently the workhorse for both clinical and research cine CMR.

## Contrast

From the early 1980's, the use of exogenous intravenously administered contrast agents had been explored in conjunction with MRI, particularly for imaging the brain. MRI contrast agents were found to improve the detection of multiple pathological abnormalities in the brain, including better depiction of solid tissue from adjacent oedema. Similar results were subsequently identified when imaging the heart with CMR. In 1982, intravenous administration of a manganese chelate contrast agent was demonstrated in differentiating ischaemic myocardium (“a dark spot”) from normal tissue in a canine model. Manganese had a limited clinical role due to its toxicity, however, other agents such as gadopentetate dimeglumine were investigated in the early 1980's. At present, gadolinium-based contrast agents remain the cornerstone of CMR to improve the depiction of multiple cardiovascular abnormalities, ranging from perfusion deficits and myocardial infarction to myocardial tumours and inflammation.

In 1989, de Roos and colleagues described the different enhancement patterns in patients with occlusive vs. reperfused infarction (19). Over the following decade, improvements in CMR technology and sequences improving the contrast between normal and infarcted myocardium, in concert with a proliferation of gadolinium-based contrast agents, led to gadolinium-enhanced MR becoming a cornerstone of CMR's clinical utility as a modality. In 2000, a seminal paper described the use of a 180 degree inversion-recovery preparatory pulse combined with a subsequent gradient echo image acquisition. In this method, normal myocardial signal relaxed to steady state at a different rate than infarcted tissue due to their difference in T1 relaxation times. Image acquisition at the inversion time when normal tissue had zero signal (the “null” point after the inversion pulse) provided a substantial improvement in contrast to noise ratio between the infarcted myocardial tissue and the adjacent normal myocardium (20). Images were acquired under steady state conditions (e.g., 10–30 min) after gadolinium contrast administration. The so-called “late gadolinium enhancement” or LGE CMR method led to a critical study by Kim et al. showing contrast-enhanced CMR could differentiate irreversible from reversible myocardial dysfunction prior to patients proceeding to percutaneous or surgical revascularisation (21).

Interpreting late-gadolinium enhanced images was extended to other pathologies, including infiltrative diseases and tumours. Patterns of mural distribution on LGE CMR images began to be described for both ischaemic and non-ischaemic cardiomyopathies. The extent of myocardial LGE appeared related to the risk of adverse cardiovascular events as well as to specific genotypes in the cases of Mendelian disease. At present, the LGE CMR method has become central to the characterisation of multiple types of acquired and genetic cardiac diseases.

## Myocardial perfusion

Myocardial perfusion evaluation by CMR was first described in 1990 also by Atkinson and colleagues. They observed the first pass kinetics of a rapid bolus of gadolinium-DTPA through the cardiac chambers and myocardium (22). This technique was subsequently augmented by the introduction of common pharmacological cardiac agents such as dipyridamole (23) and dobutamine (24) through the 1990's. Today, a vasodilator approach using adenosine or regadenoson is preferred. Further technical developments in the pulse sequence design and contrast agents have led to stress perfusion now being a key component of the assessment of coronary artery disease using CMR. Stress CMR has been demonstrated to be a feasible and efficient tool for the diagnosis of myocardial ischaemia when compared to other non-invasive modalities such as SPECT in large prospective trials (25). Current advances in stress CMR have focused on quantification of myocardial blood flow in order to supplement the qualitative interpretation of images (26).

## Quantitative CMR

The bulk of CMR interpretation relies on visual identification of varying myocardial signal intensity. However, these signals may also be quantified. Signal quantification in CMR is a technique that provides pixel-by-pixel representation of absolutely denominated numerical T1 or T2 properties, expressed in units of time (ms). These quantitative imaging approaches produce parametric maps of the entire myocardium, allowing rapid assessment of both diffuse and focal myocardial abnormalities.

The roots of parametric mapping emerged very early in the development of NMR. In particular, T1 relaxation experiments were slow, requiring many minutes (or even hours) to acquire depending on the signal magnitude. David Look and Donald Locker solved this in 1970 whilst grappling with methods to automate the challenge of calculating short T1 values (27). They published their method in 1970. Remarkably, Look and Locker only learnt of the new life their method had taken on within medical MRI in 2014 via a serendipitous email. To their surprise, the term “Look-Locker” had already been mentioned in more than 2,300 papers and 140 patents (28).

The Look-Locker technique was modified to perform measurement of myocardial T1 relaxation times within a single breath-hold by Messroghli and colleagues in 2004. Their pulse sequence, named MOLLI (MOdified Look-Locker Inversion recovery) (29), acquired an image where the signal intensity of each pixel corresponds to the T1 relaxation time of that pixel. Subsequent modifications of the original MOLLI sequence have led to the more exotic acronyms in the field of CMR such as shMOLLI, SASHA and SAPPHIRE, each representing modifications or alternatives to the original MOLLI scheme. With non-diseased myocardium having a predictable T1 relaxation time which becomes altered in the presence of pathology such as oedema, fibrosis, and infiltrative diseases, T1 mapping is now relatively routine within clinical workflows to detect focal or diffuse disease. Extensions of the approach such as extracellular myocardial volume quantification and T2 and T2* mapping also have now been incorporated into workstreams depending on the myocardial disease being studied.

## The bridge to population science

The advancements described above have catapulted CMR from the relatively esoteric to being a mainstream clinical diagnostic modality. Moreover, with its accurate quantification came the opportunity to evaluate early, asymptomatic tissue remodelling not evident with other non-invasive techniques. It was quickly apparent that CMR had a role to play in assessing subclinical cardiovascular disease and how these insights could be used in risk assessment and prognostication.

## MESA

By the 1980s, cardiovascular risk factors in the general population that led to clinical cardiovascular events were identified, most notably from work in the Framingham Study. In particular, age, gender, blood pressure and cholesterol elevation as well as diabetes were termed “traditional” risk factors that identified individuals at high risk for subsequent cardiovascular disease. Notably, most early population-based studies such as Framingham evaluated European ancestry populations, typically enrolling men. By the mid-1990s, the concept of “novel” risk factors took hold, with the premise that subclinical cardiovascular disease could be detected with new methods, and potentially modified to reduce clinical disease expression. Besides serum and genetic testing, advanced imaging methods were identified as methods to phenotype subclinical cardiovascular disease. In the United States, this led to the National Heart, Lung and Blood Institute (NHLBI)-sponsored Multi-Ethnic Study of Atherosclerosis (MESA) population-based cohort. The MESA study was also designed to reflect ethnic diversity of the United States population at that time. MESA investigators sought to maximise the statistical opportunities for risk factor association and prediction; this led to all MESA participants undergoing the same exhaustive battery of tests, often acquired over a multiple day interval.

The CMR component of MESA was led by David Bluemke and João Lima (30). More than 5,000 men and women without clinical cardiovascular disease underwent CMR examination at six university sites in the United States. Given the relative shortage of MRI scanner capacity, this represented an enormous commitment of scarce hospital resources to imaging apparently healthy individuals at a time when MRI was otherwise reserved for the most complex and in-need patients.

When the CMR MESA protocol was finalised (1999), Hawkes' SSFP cine sequences were non-standard and commercially available at only half of the CMR MESA sites. In order to provide standardisation of methods, the initial MESA CMR protocol used the better validated fast gradient-recalled echo cine imaging. MESA permitted large-scale exploration of cardiac structure and function and demonstrated its association with cardiovascular outcomes in the general population. As examples, novel insights into the importance of mass:volume ratio (31) as well as the relevance of the right ventricle (32), independent of the left ventricle, as biomarkers of subclinical cardiovascular disease in healthy individuals were novel. In 2010, the NHLBI authorised additional funding for re-examining a large portion of the original MESA cohort using CMR and other phenotypic and genotyping methods. This second CMR MESA examination added the use of gadolinium contrast to the base CMR protocol. LGE CMR contributed to understanding regarding unrecognised myocardial scar—demonstrating the strong association between scar and adverse cardiovascular events even in those without known myocardial infarction (33). In addition, gadolinium administration allowed pre- and post-contrast T1 mapping to be performed in more than 1,000 study participants.

As well as the scientific insights MESA has continued to provide since its conception a quarter of a century ago, another of its legacies is how it has allowed CMR to proliferate as the modality of choice in large-scale, prospective population studies across the world. Where MESA led, cohorts such as the Jackson Heart Study, Study of Health in Pomerania, Framingham Heart Study Offspring Cohort, Dallas Heart Study and AGES Reykjavik incorporated CMR into their population studies. The largest effort, however, was yet to come, spurred by the concept of genotype-phenotype association.

## UK Biobank

In the early 2000's, the Medical Research Council and Wellcome Trust decided to establish the UK Biobank cohort to investigate risk factors for diseases of middle and old age. Between 2,006–10, 500,000 individuals were recruited and extensive questionnaire data, physical measurements and biological samples collected. The desire to better understand the role of genetics in phenotypic and disease variation has been a major driver of the UK Biobank project and its sample size. As such, a key component has been the collection of whole-genome genetic data in every participant.

In 2009, three years since the first participants had begun being recruited, a proposal was submitted for conducting imaging assessments as an enhancement to the UK Biobank study. This was to be done in 100,000 of the 500,000 participants and that these were to be performed in regional, non-medical settings. This ambitious, perhaps even radical, plan was initially deferred by the funders but the UK Biobank imaging working group, in collaboration with international experts, returned with a bolstered case and were awarded funding for this unprecedented task. The CMR component of the imaging enhancement was led by Steffen Petersen, Stefan Piechnik and Stefan Neubauer. A pilot phase consisting of performing 5,000 examinations was successfully completed in 2014 with approval to proceed to 100,000; to date, over 60,000 participants have been imaged with funding recently agreed to additionally perform repeat examinations in 10,000 individuals.

CMR in the UK Biobank has provided many novel insights regarding associations with traditional and non-traditional cardiovascular risk factors, allowed development of novel imaging biomarkers and seen the publication of the first genome-wide association studies of CMR phenotypes (34–38). However, just as a legacy of MESA is in its inspiration of using CMR in population studies, it can be argued that the UK Biobank CMR effort too has a legacy of equal note to the science it has produced.

By 2015, the UK Biobank core lab had commenced work on manually segmenting the 5,000 pilot cases however, it was always clear that manual analysis of the intended 100,000 CMR examinations was never going to be feasible. Thus, UK Biobank provided not only the supply of ground truth but also the demand and impetus for a solution to be found. The manual segmentation by the core lab led to automated algorithms using deep learning methods for CMR analysis, first solved by Bai (39) and subsequently others (40–42). The ability to segment all four chambers, in any view, in every frame of the cardiac cycle, without any human input and all of this within seconds has been a profound legacy that the UK Biobank has left upon the field of CMR. This development led to another significant advance with the possibility of relating detailed CMR-derived imaging phenotypes to paired UK Biobank genomics, proteomics and metabolomics data.

## Conclusion

It is nearly 90 years since Bloch and Purcell discovered NMR and just over 50 since Lauterbur and Mansfield recognised its application to medical imaging. In Look's personal reflections on the sequence that bears his name, he concludes by saying “the cost of research is small, but the long-term payoff can be huge”. It is a fool's errand to predict where CMR might be 50 years hence but as long as it continues to be supported and attracts individuals willing to innovate and push boundaries, it will continue to offer new solutions to the age-old challenge of cardiovascular disease.
